# A new Kmeans clustering model and its generalization achieved by joint spectral embedding and rotation

**DOI:** 10.7717/peerj-cs.450

**Published:** 2021-03-30

**Authors:** Wenna Huang, Yong Peng, Yuan Ge, Wanzeng Kong

**Affiliations:** 1 School of Computer Science and Technology, Hangzhou Dianzi University, Hangzhou, Zhejiang, China; 2 Center for OPTical IMagery Analysis and Learning (OPTIMAL), Northwestern Polytechnical University, Xi’an, Shaanxi, China; 3 MoE Key Laboratory of Advanced Perception and Intelligent Control of High-end Equipment, Anhui Polytechnic University, Wuhu, Anhui, China; 4 Key Laboratory of Brain Machine Collaborative Intelligence of Zhejiang Province, Hangzhou, Zhejiang, China

**Keywords:** Kmeans clustering, Spectral clustering, Spectral rotation, Data similarity

## Abstract

The Kmeans clustering and spectral clustering are two popular clustering methods for grouping similar data points together according to their similarities. However, the performance of Kmeans clustering might be quite unstable due to the random initialization of the cluster centroids. Generally, spectral clustering methods employ a two-step strategy of spectral embedding and discretization postprocessing to obtain the cluster assignment, which easily lead to far deviation from true discrete solution during the postprocessing process. In this paper, based on the connection between the Kmeans clustering and spectral clustering, we propose a new Kmeans formulation by joint spectral embedding and spectral rotation which is an effective postprocessing approach to perform the discretization, termed KMSR. Further, instead of directly using the dot-product data similarity measure, we make generalization on KMSR by incorporating more advanced data similarity measures and call this generalized model as KMSR-G. An efficient optimization method is derived to solve the KMSR (KMSR-G) model objective whose complexity and convergence are provided. We conduct experiments on extensive benchmark datasets to validate the performance of our proposed models and the experimental results demonstrate that our models perform better than the related methods in most cases.

## Introduction

Clustering is one of the important research contents in many communities such as data mining and pattern recognition. Basically, it aims to group the data points into different clusters according to their similarities or densities (Ubukata, 2019; Ren, Zhang & Zhang, 2019). Over the past decades, a number of clustering algorithms have been proposed such as the *K*means clustering, spectral clustering (Ng, Jordan & Weiss, 2001), min-max cut (Ding et al., 2001; Nie et al., 2010), subspace clustering (Nie & Huang, 2016; Xie et al., 2020), and multi-view clustering (Nie, Tian & Li, 2018; Cai et al., 2013). Among the existing clustering methods, the most popular one is the *K*means clustering algorithm due to its simpleness and efficiency, which aims to learn certain cluster centroids to minimize the within cluster data distances. However, the *K*means algorithm suffers a great impact on its clustering performance due to the random initialization of cluster centroids.

By characterizing the data connection with an appropriate graph whose vertices represent the data points and the weights represent the connection between data pairs, spectral clustering tries to partition the vertices into different clusters by minimizing the cut information. There are some popular spectral clustering algorithms such as the ratio cut (RCut) (Hagen & Kahng, 1992), normalized cut (NCut) (Shi & Malik, 1997), clustering with adaptive neighbors (CAN) and its projected version (PCAN) (Nie, Wang & Huang, 2014), multiclass spectral clustering (Yu & Shi, 2003), constrained Laplacian rank (Nie et al., 2016), and nonnegative matrix factorization (Peng et al., 2018). Given a built graph, existing spectral clustering methods usually employ a two-step strategy to complete the clustering; one is performing eigen-decomposition on the graph Laplacian matrix to obtain the scaled cluster indicator matrix based on which the other aims to make discretization to get the final cluster assignment. The former step is recognized as spectral embedding and the latter step as postprocessing. Generally, the existing two approaches to complete the postprocessing task of recovering the final discrete cluster indicators from the relaxed continuous spectral vectors are the *K*means clustering and spectral rotation. As pointed by (Huang, Nie & Huang, 2013; Chen et al., 2017), using spectral rotation as the postprocessing step can usually obtain better clustering performance than that of *K*means postprocessing. However, such two-stage process has an obvious disadvantage that the final assignments may deviate far from the true discrete solution (Huang, Nie & Huang, 2013).

As mentioned above, both the *K*means clustering and the spectral clustering have limitations in respective fields. To this end, in this paper, we first derive the underlying connection between the *K*means clustering and spectral clustering, and then propose a new *K*means formulation by jointly performing spectral embedding and spectral rotation. The resultant KMSR model can effectively alleviate the drawback of the randomness in the initialization of cluster centroids of *K*means. Moreover, the two sub-objectives of spectral embedding and spectral rotation are jointly optimized, which can co-evolve to the optimum and avoid the sub-optimality caused by the two-step strategy. Due to that the KMSR is originated from the *K*means clustering, it measures the data similarity by directly using the dot-product weighting scheme whose performance is limited in dealing with complicated data sets. To accommodate more advanced graphs and then improve the performance of KMSR, we make corresponding extension on it by replacing the dot-product of data matrix with predefined graphs such as CAN and PCAN (Nie, Wang & Huang, 2014), leading to the generalized version KMSR-G. Mathematically, the KMSR model objective involves three variables respectively corresponding to the relaxed continuous cluster indicator matrix, the discrete cluster indicator, and the orthogonal transformation matrix to bridging them; therefore, under the coordinate blocking framework, we design an efficient optimization method to alternately update them, whose complexity and convergence property are also analyzed. We conduct extensive experiments on representative benchmark data sets to evaluate the performance of our proposed models. By comparing the clustering performance of KMSR and KMSR-G with related models, they both perform better in most cases.

The remainder of this paper is organized as follows. We give brief introductions to some related works including the *K*means, spectral clustering and spectral rotation in ‘Related Work’. In ‘The Proposed Model’, we first derive the model formulation of KMSR based on depicting the connection between *K*means clustering and spectral clustering, and then provide the detailed optimization process to KMSR model objective. Besides, the complexity and convergence analysis of KMSR, its generalization to KMSR-G are included. In ‘Experiment’, extensive experiments are conducted on representative benchmark data sets to evaluate the effectiveness of KMSR and KMSR-G in data clustering. ‘Conclusion’ concludes the whole paper and puts forward a future work.

**Notations**. In this paper, matrices are written as boldface uppercase letters. Vectors are written as boldface lowercase letters. For example, the (*i*, *j*)-th element of matrix **W** is *w*_*ij*_. The squared ℓ_2_-norm of matrix **W** ∈ ℝ^*n*×*m*^ is ${\mathop{\parallel \mathbf{W}\parallel }\nolimits }_{2}^{2}={\mathop{\sum }\nolimits }_{i=1}^{n}{\mathop{\sum }\nolimits }_{j=1}^{m}{w}_{ij}^{2}$. By default, we use **w**_*i*_ to represent the *i*th column of **W** and **w**^*j*^ to represent its *j*th row. We use ℝ and 𝔹 to represent the real domain and the binary domains, respectively.

## Related Works

### Kmeans clustering

Given a data matrix **X** = [**x**_1_, **x**_2_, …, **x**_*n*_] ∈ ℝ^*d*×*n*^, the *K*means clustering aims to partition **X** into *c* (1 ≤ *c* ≤ *n*) clusters *C* = [*C*_1_, *C*_2_, …, *C*_*c*_] such that the within-cluster sum of squared distances can be minimized and the sum of squared distances between clusters can be maximized. Mathematically, the objective function of *K*means clustering is

(1) \begin{eqnarray*}\min _{C}\sum _{i=1}^{c}\sum _{{\mathbf{x}}_{j}\in {C}_{i}}{ \left\| {\mathbf{x}}_{j}-{\mu }_{i} \right\| }_{2}^{2},\end{eqnarray*}

where *μ*_*i*_ is the centroid corresponding to the cluster *C*_*i*_. To optimize objective Eq. (1), the membership of each data point and the centroid of each cluster are alternately updated.

### Spectral clustering

For spectral clustering, we first need to construct a graph affinity matrix **A** ∈ ℝ^*n*×*n*^ according to certain similarity measures to depict the connection between data pairs. Let ${\mathbf{y}}^{i}{\mathop{{|}}\nolimits }_{i=1}^{n}$ be the *i*th row vector of matrix **Y** = [**y**^1^; **y**^2^; ⋯; **y**^*n*^] ∈ 𝔹^*n*×*c*^, which corresponds to the cluster indicator vector for **x**_*i*_. The *j*th element of **y**^*i*^ is 1 if **x**_*i*_ ∈ *C*_*j*_, and 0 otherwise. By defining the scaled cluster indicator matrix **F** as **F** = **Y**(**Y**^*T*^**Y**)^−1∕2^ whose *j*th column is given by

(2) \begin{eqnarray*}{\mathbf{f}}_{j}=[\underbrace{0,\ldots ,0{}}_{\sum _{i=1}^{j-1}{n}_{i}},\underbrace{1,\ldots ,1{}}_{{n}_{j}},\underbrace{0,\ldots ,0{}}_{\sum _{i=j+1}^{c}{n}_{i}}]^{T}/\sqrt{{n}_{j}},\end{eqnarray*}

where *n*_*j*_ is the number of data in the *j*th cluster. Then, the objective function of spectral clustering can be formulated as

(3) \begin{eqnarray*}\min _{\mathbf{F}}\text{Tr}({\mathbf{F}}^{T}\mathbf{L}\mathbf{F}), s.t.\mathbf{F}=\mathbf{Y }({\mathbf{Y }}^{T}\mathbf{Y })^{-1/2}.\end{eqnarray*}

Here **L** = **D** − **A** is the Laplacian matrix, where **D** is the diagonal degree matrix with its *i*th diagonal element defined as ${d}_{ii}={\mathop{\sum }\nolimits }_{j=1}^{n}{a}_{ij}$.

Since **F**^*T*^**F** = (**Y**^*T*^**Y**)^−1∕2^**Y**^*T*^**Y**(**Y**^*T*^**Y**)^−1∕2^ = **I**, the embedding **F** can be obtained by stacking the eigenvectors of **L** corresponding to its *c* smallest eigen-values. However, **F** is a real-valued matrix and therefore a postprocessing step such as the *K*means clustering or spectral rotation (Huang, Nie & Huang, 2013) is necessary to perform discretization.

It is easy to find that the solution to Eq. (3) is not unique. That is, for any solution **F**, **FR** is another solution where **R** is an arbitrary orthogonal matrix. Therefore, spectral rotation aims at finding a proper orthogonal and normalized **R** such that the resultant **FR** are closer to the discrete indicator matrix solution set than the **F** in *K*means. Mathematically, it aims to minimize the following objective

(4) \begin{eqnarray*}\min _{\mathbf{Y },\mathbf{R}}{\mathop{\parallel \mathbf{FR}-\mathbf{Y }\parallel }\nolimits }_{2}^{2}, s.t.\mathbf{Y }\in {\mathbb{B}}^{n\times c},{\mathbf{Y 1}}_{c}={\mathbf{1}}_{n},{\mathbf{R}}^{T}\mathbf{R}=\mathbf{I},\end{eqnarray*}

where **1**_*c*_ and **1**_*n*_ are both all-one column vectors with sizes of *c* × 1 and *n* × 1, respectively. By using the alternative optimization method, objective Eq. (4) can be solved and therefore the final cluster assignment can be obtained.

## The Proposed Model

In this section, we formulate the model objective function of KMSR and derive its optimization method. Besides, the complexity and convergence analysis are provided.

### Model formulation

By introducing two matrices **U** = [*μ*_1_, *μ*_2_, …, *μ*_*c*_] ∈ ℝ^*d*×*c*^ and **Y** such that (**Y** ∈ 𝔹^*n*×*c*^, **Y 1**_*c*_ = **1**_*n*_) to respectively represent the cluster centroids and indices, the *K*means objective in Eq. (1) can be reformulated as

(5) \begin{eqnarray*}\min _{\mathbf{Y },\mathbf{U}}\parallel \mathbf{X}-{\mathbf{UY }}^{T}{\parallel }^{2}\Leftrightarrow \min _{\mathbf{ Y },\mathbf{U}}\text{Tr}({\mathbf{Y U}}^{T}{\mathbf{UY }}^{T})-2\text{Tr}({\mathbf{X}}^{T}{\mathbf{UY }}^{T}).\end{eqnarray*}

Since the solution to **U** is **U** = **XY**(**Y**^*T*^**Y**)^−1^, we have Tr(**Y U**^*T*^**UY**^*T*^) = Tr(**X**^*T*^**UY**^*T*^) and then Eq. (5) can be written as

(6) \begin{eqnarray*}\min _{\mathbf{Y },\mathbf{U}}-\text{Tr}({\mathbf{X}}^{T}{\mathbf{UY }}^{T})\Leftrightarrow \min _{\mathbf{ Y }}-\text{Tr}(({\mathbf{Y }}^{T}\mathbf{Y })^{- \frac{1}{2} }{\mathbf{Y }}^{T}({\mathbf{X}}^{T}\mathbf{X})\mathbf{Y }({\mathbf{Y }}^{T}\mathbf{Y })^{- \frac{1}{2} })\nonumber\\\displaystyle \Leftrightarrow \min _{\mathbf{F}}-\text{Tr}({\mathbf{F}}^{T}({\mathbf{X}}^{T}\mathbf{X})\mathbf{F}),\end{eqnarray*}

where **F** ∈ ℝ^*n*×*c*^ and $\mathbf{F}\triangleq \mathbf{Y }({\mathbf{Y }}^{T}\mathbf{Y })^{- \frac{1}{2} }$.

By checking Eq. (6), an usual way to solve it is relaxing the binary matrix **F** to real domain but keeping its orthogonality intact. Then, we obtain the following formulation

(7) \begin{eqnarray*}\min _{\mathbf{F}}-\text{Tr}({\mathbf{F}}^{T}{\mathbf{X}}^{T}\mathbf{X}\mathbf{F}), s.t.{\mathbf{F}}^{T}\mathbf{F}=\mathbf{I},\mathbf{F}\in {\mathbb{R}}^{n\times c}.\end{eqnarray*}

Note that **F** in Eq. (7) is the continuous relaxation which preserves the orthogonality but misses the discrete nature of **Y**. If we use *K*means to find the cluster assignment which aims to jointly find the cluster indicator matrix **Y** and centroids **C** by ${\min }_{\mathbf{Y }\in {\mathbb{B}}^{n\times c},{\mathbf{Y 1}}_{c}={\mathbf{1}}_{n},\mathbf{C}}{\mathop{\parallel \mathbf{F}-\mathbf{Y C}\parallel }\nolimits }_{F}^{2}$, it can only guarantee that **Y C** best approximates the relaxed continuous vector matrix **F** and cannot guarantee that such yielded **Y** best approximates **Y**. As mentioned in ‘Spectral Clustering’, the relaxed solution to Eq. (7) is not unique. Actually, for any solution **F**, **FQ** is another solution where **Q** is an arbitrary orthonormal matrix. The goal of spectral rotation is to find a proper **Q** such that the resulting **FQ** are closer to the discrete indicator matrix solution set than the **F** in *K*means. Therefore, we can take the idea of spectral rotation into account to perform post-processing operation on the optimal **F**^∗^ of Eq. (7) to obtain the final cluster indicator matrix.

Inspired by Chen et al. (2017), we aim at finding an orthonormal matrix **Q** ∈ ℝ^*c*×*c*^ to minimize the discrepancy between $\mathbf{Y }({\mathbf{Y }}^{T}\mathbf{Y })^{- \frac{1}{2} }$ and **F**^∗^**Q** as the postprocessing step. Mathematically, it can be achieved by solving the following objective

(8) \begin{eqnarray*}\min _{\mathbf{Y }\in {\mathbb{B}}^{n\times c},\mathbf{Q}}{\mathop{\parallel \mathbf{Y }({\mathbf{Y }}^{T}\mathbf{Y })^{- \frac{1}{2} }-{\mathbf{F}}^{\ast }\mathbf{Q} \parallel }\nolimits }_{F}^{2}, s.t.{\mathbf{Q}}^{T}\mathbf{Q}=\mathbf{I},\mathbf{Q}\in {\mathbb{R}}^{c\times c}.\end{eqnarray*}

From the above analysis, we realize that an intuitive way to handle a spectral clustering task is to get **F**^∗^ by solving Eq. (7) followed by an appropriate **Q** from Eq. (8) to finally obtain the final cluster indicator matrix **Y**. To avoid the sub-optimality caused by such two-step process, in this paper, we propose to jointly optimize the objectives of Eq. (7) and Eq. (8) which respectively correspond to the spectral embedding and rotation, leading to the following new *K*means formulation (termed **KMSR**) as

(9) \begin{eqnarray*}\min _{\mathbf{Y },\mathbf{F},\mathbf{Q}}-\text{Tr}({\mathbf{F}}^{T}{\mathbf{X}}^{T}\mathbf{XF})+\lambda {\mathop{\parallel \mathbf{Y }({\mathbf{Y }}^{T}\mathbf{Y })^{- \frac{1}{2} }-\mathbf{FQ}\parallel }\nolimits }_{F}^{2}, s.t.\mathbf{Y }\in {\mathbb{B}}^{n\times c},{\mathbf{Y 1}}_{c}={\mathbf{1}}_{n},\nonumber\\\displaystyle {\mathbf{F}}^{T}\mathbf{F}=\mathbf{I},{\mathbf{Q}}^{T}\mathbf{Q}=\mathbf{I},\end{eqnarray*}

where *λ* > 0 is a regularization parameter to control the balance between the two items.

In spectral clustering, the normalized Laplacian matrix **L**_*n*_ is defined as

(10) \begin{eqnarray*}{\mathbf{L}}_{n}=\mathbf{I}-{\mathbf{D}}^{-1/2}\mathbf{A}{\mathbf{D}}^{-1/2}.\end{eqnarray*}

If we replace **L** in Eq. (3) with **L**_*n*_, it becomes the objective function of the normalized cut. Since Tr(**F**^*T*^**F**) is a constant, we can find an interesting point that there exists an equivalence between the *K*means clustering and the normalized cut. That is, the graph affinity matrix in *K*means clustering employs the simple dot-product weighting scheme, i.e., **X**^*T*^**X**, while it is $\tilde {\mathbf{A}}\triangleq {\mathbf{D}}^{-1/2}\mathbf{A}{\mathbf{D}}^{-1/2}$ in normalized cut.

We know that the graph quality plays an important role in spectral clustering. Sometimes, we need to learn a more robust graph to characterize the connection among data points than simply constructing it by fixed rules. Therefore, to further enhance the model performance, we make the generalization on KMSR by introducing $\tilde {\mathbf{A}}$ as the graph matrix. That is, we can incorporate more advanced graphs instead of the simple dot-product weighting scheme. We name the generalized model as **KMSR-G** which has the following objective function

(11) \begin{eqnarray*}\min _{\mathbf{Y },\mathbf{F},\mathbf{Q}}-\text{Tr}({\mathbf{F}}^{T}\tilde {\mathbf{A}}\mathbf{F})+\lambda {\mathop{\parallel \mathbf{Y }({\mathbf{Y }}^{T}\mathbf{Y })^{- \frac{1}{2} }-\mathbf{FQ}\parallel }\nolimits }_{2}^{2}, s.t.\mathbf{Y }\in {\mathbb{B}}^{n\times c},{\mathbf{Y 1}}_{c}={\mathbf{1}}_{n},{\mathbf{F}}^{T}\mathbf{F}=\mathbf{I},{\mathbf{Q}}^{T}\mathbf{Q}=\mathbf{I}.\end{eqnarray*}

Obviously, KMSR-G is a general model to accommodate any predefined (or pre-learned) graph, which is expected to achieve better performance than KMSR especially when handling complicated data sets.

**Figure 1:**
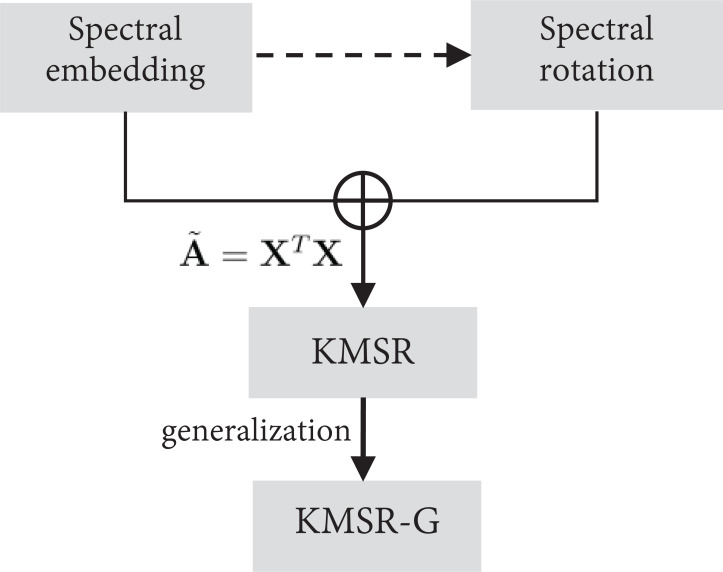
The framework of the proposed models, KMSR and KMSR-G.

Figure 1 intuitively shows the framework of our proposed models from which we can observe that KMSR jointly performs spectral embedding and rotation on a specified graph. Further, KMSR-G is a generalized model to accommodate other advanced graphs, leading to better clustering performance.

### Model optimization

The only difference between the model objectives of KMSR in Eq. (9) and KMSR-G Eq. (11) is the graph affinity matrix; therefore, they share the identical optimization procedure. Below taking the KMSR-G as an example, we show its detailed optimization steps based on the alternating framework (Tang et al., 2020). That is, we update one variable by fixing the others.

■ Update **Q** with **Y** and **F** fixed. The sub-objective associated with **Q** is

(12) \begin{eqnarray*}\min _{\mathbf{Q}}{\mathop{\parallel \mathbf{Y }({\mathbf{Y }}^{T}\mathbf{Y })^{- \frac{1}{2} }-\mathbf{FQ} \parallel }\nolimits }_{2}^{2}, s.t.{\mathbf{Q}}^{T}\mathbf{Q}={\mathbf{I}}_{c},\mathbf{Q}\in {\mathbb{R}}^{c\times c},\end{eqnarray*}

which can be further reformulated into

(13) \begin{eqnarray*}\max _{\mathbf{Q}}\text{Tr}(({\mathbf{Y }}^{T}\mathbf{Y })^{- \frac{1}{2} }{\mathbf{Y }}^{T}\mathbf{FQ}), s.t.{\mathbf{Q}}^{T}\mathbf{Q}={\mathbf{I}}_{c},\mathbf{Q}\in {\mathbb{R}}^{c\times c}.\end{eqnarray*}

By denoting ${\mathbf{M}}_{2}\triangleq ({\mathbf{Y }}^{T}\mathbf{Y })^{- \frac{1}{2} }{\mathbf{Y }}^{T}\mathbf{F}$, objective Eq. (13) can be converted to

(14) \begin{eqnarray*}\max _{\mathbf{Q}}\text{Tr}({\mathbf{M}}_{2}\mathbf{Q}), s.t.{\mathbf{Q}}^{T}\mathbf{Q}={\mathbf{I}}_{c},\mathbf{Q}\in {\mathbb{R}}^{c\times c}\end{eqnarray*}

Suppose that the singular value decomposition of **M**_2_ is **M**_2_ = **U**Σ**V**^*T*^ and then we have

(15) \begin{eqnarray*}\text{Tr}({\mathbf{M}}_{2}\mathbf{Q})=\text{Tr}(\mathbf{QU}\Sigma {\mathbf{V }}^{T})=\text{Tr}(\Sigma \mathbf{E})=\sum _{i=1}^{c}{\lambda }_{ii}{e}_{ii},\end{eqnarray*}

where **E** = **V**^*T*^**QU** with *λ*_*ii*_ and *e*_*ii*_ as the (*i*, *i*)-th elements of matrix Σ and **E**, respectively.

Since **E**^*T*^**E** = **U**^*T*^**Q**^*T*^**V V**^*T*^**QU** = **I**_*c*_, i.e., ${\mathop{\sum }\nolimits }_{j=1}^{c}{e}_{ji}^{2}=1$, we know *e*_*ii*_ ≤ 1 (1 ≤ *i* ≤ *c*). Meanwhile, *λ*_*ii*_ is non-negative since it is a singular value. Therefore, we have $\text{Tr}({\mathbf{M}}_{2}\mathbf{Q})={\mathop{\sum }\nolimits }_{i=1}^{c}{\lambda }_{ii}{e}_{ii}\leq {\mathop{\sum }\nolimits }_{i=1}^{c}{\lambda }_{ii}$, and the equality holds when *e*_*ii*_ = 1 (1 ≤ *i* ≤ *c*). That is to say, Tr(**M**_2_**Q**) reaches its maximum when **E** = **I**_*c*_ = **V**^*T*^**QU**. Then we obtain the optimal solution of **Q** as

(16) \begin{eqnarray*}\mathbf{Q}={\mathbf{V U}}^{T}.\end{eqnarray*}

■ Update **F** with **Y** and **Q** fixed. The sub-objective associated with **F** is

(17) \begin{eqnarray*}\min _{\mathbf{F}}-\text{Tr}({\mathbf{F}}^{T}\tilde {\mathbf{A}}\mathbf{F})+\lambda \text{Tr}(\mathbf{FQ}{\mathbf{Q}}^{T}{\mathbf{F}}^{T}) -2\lambda \text{Tr}({\mathbf{F}}^{T}{\mathbf{M}}_{1}{\mathbf{Q}}^{T}), s.t.{\mathbf{F}}^{T}\mathbf{F}={\mathbf{I}}_{c},\mathbf{F}\in {\mathbb{R}}^{n\times c},\end{eqnarray*}

where symmetric matric $\tilde {\mathbf{A}}\in {\mathbb{R}}^{n\times n}$ and ${\mathbf{M}}_{1}\triangleq \mathbf{Y }({\mathbf{Y }}^{T}\mathbf{Y })^{- \frac{1}{2} }\in {\mathbb{R}}^{n\times c}$. Since **Q** ∈ ℝ^*c*×*c*^ is a square matrix and **F**^*T*^**F** = **I**_*c*_, the second term in objective Eq. (17) is a constant and then we can get the simplified version of objective Eq. (17) as

(18) \begin{eqnarray*}\min _{\mathbf{F}}-\text{Tr}({\mathbf{F}}^{T}\tilde {\mathbf{A}}\mathbf{F})-2\lambda \text{Tr}({\mathbf{F}}^{T}{\mathbf{M}}_{1}{\mathbf{Q}}^{T}), s.t.{\mathbf{F}}^{T}\mathbf{F}={\mathbf{I}}_{c},\mathbf{F}\in {\mathbb{R}}^{n\times c}.\end{eqnarray*}

Denoting **B**≜**M**_1_**Q**^*T*^, we have

(19) \begin{eqnarray*}\max _{\mathbf{F}}\text{Tr}({\mathbf{F}}^{T}\tilde {\mathbf{A}}\mathbf{F})+2\lambda \text{Tr}({\mathbf{F}}^{T}\mathbf{B}), s.t.{\mathbf{F}}^{T}\mathbf{F}={\mathbf{I}}_{c},\mathbf{F}\in {\mathbb{R}}^{n\times c}.\end{eqnarray*}

The corresponding Lagrangian function of problem Eq. (19) is

(20) \begin{eqnarray*}\mathcal{L}(\mathbf{F},\Lambda )=\text{Tr}({\mathbf{F}}^{T}\tilde {\mathbf{A}}\mathbf{F})+2\lambda \text{Tr}({\mathbf{F}}^{T}\mathbf{B})-\text{Tr}(\Lambda ({\mathbf{F}}^{T}\mathbf{F}-{\mathbf{I}}_{c})),\end{eqnarray*}

where Λ is a Lagrangian multiplier in matrix form. Then we can obtain the KKT condition as

(21) \begin{eqnarray*} \frac{\partial \mathcal{L}}{\partial \mathbf{F}} =2\tilde {\mathbf{A}}\mathbf{F}+2\lambda \mathbf{B}-2\mathbf{F}\Lambda =\mathbf{0}\end{eqnarray*}

which is difficult to solve directly.

Essentially, problem Eq. (19) is a relaxed form of quadratic optimization problem on the Stiefel manifold (QPSM). In optimization theory, the standard form of QPSM is min_**P**^*T*^**P**=**I**_*k*__Tr(**P**^*T*^**HP** − 2**P**^*T*^**K**), where **P** ∈ ℝ^*m*×*k*^, **K** ∈ ℝ^*m*×*k*^, and the symmetric matrix **H** ∈ ℝ^*m*×*m*^. This objective can be relaxed into ${\max }_{{\mathbf{P}}^{T}\mathbf{P}={\mathbf{I}}_{k}}\text{Tr}({\mathbf{P}}^{T}\tilde {\mathbf{H}}\mathbf{P})+2\text{Tr}({\mathbf{P}}^{T}\mathbf{K})$ by introducing $\tilde {\mathbf{H}}=\alpha {\mathbf{I}}_{m}-\mathbf{H}\in {\mathbb{R}}^{m\times m}$, which is equivalent to Eq. (19). Inspired by the work (Nie, Zhang & Li, 2017), we employ the generalized power iteration (GPI) method to optimize Eq. (19) and summarize the detailed procedure in Algorithm 1.

■ Update **Y** with **F** and **Q** fixed. Similar to the optimization process of updating **Q**, the sub-objective associated with **Y** is

(22) \begin{eqnarray*}\min _{\mathbf{Y }}{\mathop{\parallel \mathbf{Y }({\mathbf{Y }}^{T}\mathbf{Y })^{- \frac{1}{2} }-\mathbf{FQ} \parallel }\nolimits }_{2}^{2}, s.t.{\mathbf{Y 1}}_{c}={\mathbf{1}}_{n},\mathbf{Y }\in {\mathbb{B}}^{n\times c}.\end{eqnarray*}

Denote **G**≜**FQ** and then optimizing Eq. (22) is equivalent to optimizing the following one

(23) \begin{eqnarray*}\max _{\mathbf{Y }}\text{Tr}(\mathbf{Y }({\mathbf{Y }}^{T}\mathbf{Y })^{- \frac{1}{2} }{\mathbf{G}}^{T}), s.t.{\mathbf{Y 1}}_{c}={\mathbf{1}}_{n},\mathbf{Y }\in {\mathbb{B}}^{n\times c}.\end{eqnarray*}

Motivated by Chen et al. (2018), objective Eq. (23) can be represented as

(24) \begin{eqnarray*}\max _{{\mathbf{Y 1}}_{c}={\mathbf{1}}_{n},\mathbf{Y }\in {\mathbb{B}}^{n\times c}}\sum _{j=1}^{c} \frac{{{\mathbf{y}}_{j}}^{T}{\mathbf{g}}_{j}}{\sqrt{{{\mathbf{y}}_{j}}^{T}{\mathbf{y}}_{j}}} .\end{eqnarray*}

Since $\sqrt{{\mathbf{y}}_{j}^{T}{\mathbf{y}}_{j}}$ involves all rows of **Y**, we can sove **Y** row-wisely; that is, we can update one row of **Y** by fixing the others as constants. Suppose we have obtained the optimal solution $\overline{\mathbf{Y }}$ in the last iteration and the corresponding objective function values is ${\mathcal{J}}^{old}(\bar {\mathbf{Y }})$. The elements of each row vector **y**^*i*^ are composed of 1 or 0 where the unique 1 indicates the cluster membership of *i*th data point. To solve the *i*th row **y**^*i*^, we only need to consider the increment of the objective function value from *y*_*ij*_ = 0 to *y*_*ij*_ = 1. The increment as can be calculated as follows

**Figure 2:**
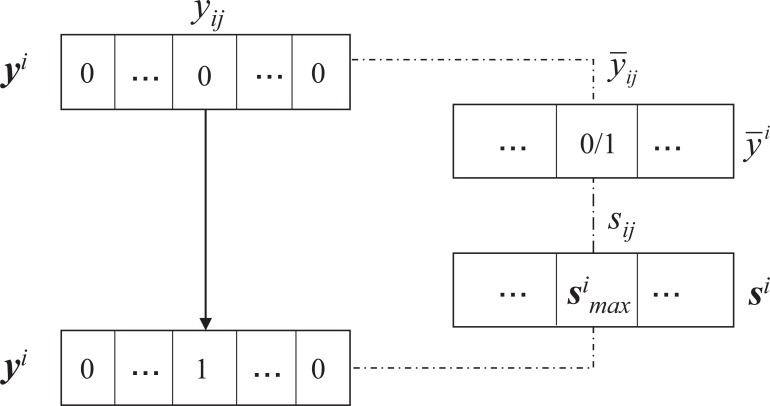
The illustration procedure of updating Y (Chen et al., 2017).

(25) \begin{eqnarray*}{s}_{ij}= \frac{{\overline{{\mathbf{y}}_{j}}}^{T}{\mathbf{g}}_{j} +{g}_{ij}(1-{\overline{y}}_{ij})}{\sqrt{{{\overline{\mathbf{y}}}_{j}}^{T}{\overline{\mathbf{y}}}_{j} +(1-{\overline{y}}_{ij})}} - \frac{{{\overline{\mathbf{y}}}_{j}}^{T}{\mathbf{g}}_{j} -{\overline{y}}_{ij}{g}_{ij}}{\sqrt{{{\overline{\mathbf{y}}}_{j}}^{T}{\overline{\mathbf{y}}}_{j} -{\overline{y}}_{ij}}} ,\end{eqnarray*}

whose graphical illustration is given in Fig. 2.

Then it can be verified that the optimal solution of **y**^*i*^ is

(26) \begin{eqnarray*}{y}_{ij}=\langle j=\arg \nolimits \max _{{l}^{{^{\prime}}}\in [1,c]}{s}_{i{l}^{{^{\prime}}}}\rangle \end{eqnarray*}

where 〈⋅〉 is 1 if the argument is true or 0 otherwise and *s*_*ij*_ is defined by Eq. (25).

As a whole, we summarize the complete procedure of solving the objective function Eq. (11) of KMSR-G in Algorithm 2.

### Model complexity and convergence analysis

In terms of the computational complexity, if ignoring the special process in graph construction in KMSR-G, KMSR and KMSR-G share similar complexities because they involve the same optimization procedure. The below complexity analysis is based on Algorithm 2.

• *Updating the variable* **F****. We use the generalized power iteration method to update **F** in Eq. (19). According to the analysis in Nie, Zhang & Li (2017), the complexity of updating **F** is $\mathcal{O}({n}^{2}c)$.
• *Updating the variable* **Q****. The complexity of updating **Q** mainly comes from the singular value decomposition of **M**_2_ ∈ ℝ^*c*×*c*^, which has the complexity of $\mathcal{O}({c}^{3})$.
• *Updating the variable* **Y****. We need $\mathcal{O}(nc)$ time to obtain **Y** because we deal with **Y** row by row. What’s more, ${\overline{{\mathbf{y}}_{j}}}^{T}\overline{{\mathbf{y}}_{j}}$ and ${\overline{{\mathbf{y}}_{j}}}^{T}{\mathbf{g}}_{j}$ can be calculated before solving **Y** and updated after solving **y**^*i*^ according to Eq. (26).

Assuming that *T*_1_ is the maximum number of iterations for the KMSR-G, *r*_1_ and *r*_2_ are the average numbers of iterations to update **F** and **Y** respectively, the overall computational complexity of KMSR-G is $\mathcal{O}({T}_{1}({n}^{2}c{r}_{1}+{c}^{3}+nc{r}_{2}))$. In general cases, we have *c* < *T*_1_ <  < *n*. In comparison with spectral clustering methods which usually have the time complexity of $\mathcal{O}({n}^{3})$ (*n* is the number of samples), we can easily find that KMSR and KMSR-G have a lower computational complexity in dealing with large-sized data sets.

Obviously, KMSR and KMSR-G have similar convergence properties due to the identical optimization procedure. Here we also give the analysis based on Algorithm 2. When solving the variable **F**, the GPI method is utilized whose optimization procedure is summarized in Algorithm 1. According to the appendix of Nie, Zhang & Li (2017), we know that the GPI method converges to a global minimum of the quadratic problem on the Stiefel manifold, which guarantees the convergence of Algorithm 1 in updating **F**. When updating the variable **Q**, the analytical solution to **Q** can be obtained based on the singular value decomposition. For the variable **Y**, we propose to optimize it in a row-by-row manner according to Eq. (26) because determining the membership of each sample is independent. So we can convert the updating of **Y** into *n* independent subproblems. Further, since each row of **Y** is a binary vector in one-hot encoding, there are finite candidate solutions for each subproblem. Therefore, it can be proved that each subproblem must have an optimal solution, which guarantees the convergence of the updating of **Y**. In total, we can come to a conclusion that the optimization of KMSR-G is expected to converge in terms of iterations.

### Discussions

We can find that the only difference between KMSR and KMSR-G is the different ways to represent the similarity matrix (also called the affinity matrix) in respective objective functions. If the normalized graph affinity matrix $\tilde {\mathbf{A}}$ is represented as **X**^*T*^**X**, KMSR-G will degenerate to KMSR. It is obvious that the clustering performance heavily depends on the quality of the input data graph in graph-based clustering. In the section of experiments, we will adopt one rule-based method (‘Heatkernel’ weighting scheme) and two learning-based methods (CAN and PCAN (Nie, Wang & Huang, 2014)) to obtain three different affinity matrices. Then we will see how much the graph quality influences the performance of the KMSR-G.

Therefore, there are two proposed models KMSR and KMSR-G in this paper and the latter can be seen as the generalization of the former. As a summary, below we summarize the main contributions of this paper.

• We propose a novel *K*means formulation (termed KMSR) by exploring the underlying equivalence between the *K*means clustering and the spectral clustering, which is finally achieved by jointly performing the spectral embedding and rotation. Mathematically, the objective of KMSR consists of two items, which respectively aim to calculate the scaled cluster indicator matrix and perform discretization to obtain the final discrete cluster indicator matrix. When compared with *K*means clustering, the randomness would be effectively alleviated in KMSR because it jointly searches for an optimal rotator in discretization process.
• By investigating the connection between the graph affinity matrices respectively employed in KMSR and spectral clustering, we make generalization on the KMSR model to make it accommodate more advanced graphs, and finally formulate the KMSR-G model. KMSR-G is also a unified model for jointly completing the spectral embedding and rotation steps, which is often expected to obtain superior performance to KMSR.
• We propose an efficient algorithm to optimize the objective function of KMSR-G (also KMSR since they share the same optimization procedure). In the iterative procedure, there are three blocks respectively corresponding to the three variables (i.e., **F**, **Q** and **Y**) involved in KMSR-G. They are co-optimized toward the optimum. Besides, we provide detailed computational complexity and convergence analysis to the optimization algorithm.
• To evaluate the performance of KMSR and KMSR-G in data clustering, we conduct extensive experiments on twelve representative benchmark data sets. The experimental results show that both KMSR abd KMSR-G perform better than the closely related counterparts.

## Experiments

In this section, we conduct experiments on twelve representative benchmark data sets to evaluate the performance of the proposed KMSR and KMSR-G models in data clustering.

### Evaluation metrics

To evaluate the clustering results, we compare the obtained label of each sample with the label provided by the data set. We use three popular metrics, i.e., *Accuracy* (Acc) , *Normalized Mutual Information* (NMI) and *Purity* (Huang, Nie & Huang, 2015) to measure the clustering performance of different models. Below we give the definition of these three metrics in turn.

Given a data point **x**_*i*_, we use *r*_*i*_ to denote the obtained cluster label and *s*_*i*_ to denote the ground-truth label provided by the data set. Then Acc is defined as

(27) \begin{eqnarray*}\text{Acc}= \frac{1}{n} \sum _{i=1}^{n}\delta ({s}_{i},\text{map}({r}_{i})),\end{eqnarray*}

where *n* is the sample size, *δ*(*x*, *y*) is the indicator function that equals to one if *x* = *y* and zero otherwise. map(*r*_*i*_) is the permutation mapping function which maps each cluster label *r*_*i*_ to the equivalent class label from the data set.

Let *C* denote the set of clusters obtained from the ground truth and *C*′ denote the set of the clusters obtained from the given model. Then mutual information MI(*C*, *C*′) is defined as

(28) \begin{eqnarray*}\text{MI}(C,{C}^{{^{\prime}}})=\sum _{{c}_{i}\in C,{c}_{j}^{{^{\prime}}}\in {C}^{{^{\prime}}}}p({c}_{i},{c}_{j}^{{^{\prime}}})\cdot \log \nolimits \frac{p({c}_{i},{c}_{j}^{{^{\prime}}})}{p({c}_{i})p({c}_{j}^{{^{\prime}}})} ,\end{eqnarray*}

where *p*(*c*_*i*_) and $p({c}_{j}^{{^{\prime}}})$ denote the probabilities that a sample arbitrarily selected from the data set belongs to the clusters *c*_*i*_ and ${c}_{j}^{{^{\prime}}}$, respectively. Besides, $p({c}_{i},{c}_{j}^{{^{\prime}}})$ is the joint probability that the selected sample belongs to the clusters *c*_*i*_ and ${c}_{j}^{{^{\prime}}}$ simultaneously. So the NMI is given as follows

(29) \begin{eqnarray*}\text{NMI}(C,{C}^{{^{\prime}}})= \frac{\text{MI}(C,{C}^{{^{\prime}}})}{\max \nolimits (H(C),H({C}^{{^{\prime}}}))} ,\end{eqnarray*}

where *H*(*C*) and *H*(*C*′) denote the entropies of *C* and *C*′ respectively.

To compute the purity, each cluster is assigned to the class which is most frequent in the cluster, and then the accuracy of this assignment is measured by counting the number of correctly assigned documents and dividing by *n*. Then the clustering Purity metric is estimated by

(30) \begin{eqnarray*}\text{Purity}= \frac{1}{n} \sum _{i=1}^{c}\max _{j}({n}_{i}^{j})\end{eqnarray*}

where *c* is the number of the clusters and *n* is the total number of the data points, ${n}_{i}^{j}$ is the number of *i*th input class that is assigned to the *j*th cluster.

It is easy to check that all Acc, NMI and Purity metrics range from zero to one and a higher value indicates a better clustering result.

### Data sets and experimental settings

Twelve real-world data sets were used in the following experiments including nine image data sets (COIL20, umist, AT&T, YaleB, Yale, PIE, AR, MNIST and jaffe) and three non-image data sets (ecoli, abalone and scale). Their basic characteristics of the sample size, dimensionality, the number of clusters were summarized in Table 1.

**Table 1:** The basic characteristics the twelve data sets used in the experiments.

Data Sets	# Samples	# Dimensions	# Clusters
ecoli	327	7	5
abalone	4177	8	3
scale	625	4	3
COIL20	1440	1024	20
umist	575	644	20
AT&T	400	189	40
YaleB	2414	1024	38
Yale	165	105	15
PIE	1428	1024	68
AR	1200	261	100
MNIST	1000	784	10
jaffe	212	177	7

The following experiments can be divided into two parts. In the former part, we aim to demonstrate the effectiveness of the KMSR model in comparison with the traditional one; In the latter part, we want to evaluate the effectiveness of the generalized KMSR-G model and also provide some insights to the influence of graph quality on the clustering performance.

**PART 1. Experimental settings to evaluate the performance of the KMSR model**. To investigate the effectiveness of the KMSR model, we perform the pairwise comparison between the KMSR and the traditional *K*means clustering. Since the *K*means clustering is sensitive to the initialization, we independently repeat it 50 times. For our KMSR model, we repeat it 20 times. The number of clusters is set as the ground-truth. Since there is a free regularization parameter in the proposed KMSR model, we tune it from candidate values $ \left\{ 1{0}^{-3},1{0}^{-2},\ldots ,1{0}^{3} \right\} $ to let it achieve the best results.

**PART 2. Experimental settings to evaluate the performance of the KMSR-G model**. First, we compare the generalized model KMSR-G with two closely related spectral clustering algorithms, NCut and RCut, to evaluate its effectiveness. Two commonly used post-processing methods (i.e., *K*means and spectral rotation) are adopted in spectral clustering; therefore, we implement this two versions of NCut respectively named as NCut+KM and NCut+SR. Similarly, we have two corresponding versions of RCut, RCut+KM and RCut+SR. The affinity matrix referred in these models is constructed by the ‘Heatkernel’ function in which the number of the nearest neighbors is set as five, and the bandwidth parameter is set as one. Second, by taking the two more advanced learning-based graph affinity matrices, CAN and PCAN, as the input graphs to KMSR-G, we respectively obtain two variant models termed KMSR-GC and KMSR-GPC. The neighborhood parameters *k* required in CAN and PCAN are tuned from $ \left\{ 5,10,15,20 \right\} $. The CAN and PCAN method are repeated only once since their clustering results are stable (Nie, Wang & Huang, 2014). Both KMSR-GC and KMSR-GPC are repeated 20 times. We use CAN and PCAN as graph learning methods in our experiments for two reasons. One is that both are joint models for graph construction and scaled cluster indicator matrix learning, leading to superior graph quality. The other is that they can adaptively determine the number of neighbors in graph construction.

For all the above mentioned models, the number of clusters is set as the ground-truth in each data set and the clustering performance is evaluated based on the metrics of Acc, NMI and Purity. Besides, we respectively set the maximum numbers of iterations in updating **F** and **Y** in our proposed models to be 50 and 10. For the free regularization parameters in related models, they are tuned from $ \left\{ 1{0}^{-3},1{0}^{-2},\ldots ,1{0}^{2},1{0}^{3} \right\} $ to let the models achieve their best results. The average clustering results and standard deviations are reported for comparison.

### Experimental results and analysis

Here we show the experimental results based on which we provide the corresponding analysis.

**PART 1**. The experimental results of the pairwise comparison between *K*means and KMSR are shown in Table 2, which includes the average results and standard deviations over multiple runs. From this table, we can find that the best results highlighted in boldface are all from the proposed KMSR model. Therefore, we can conclude that KMSR significantly outperforms the *K*means on all the used data sets in terms of all the three clustering performance evaluation metrics, which indicates the effectiveness of jointly performing spectral embedding and rotation.

**Table 2:** Clustering performance (%) of *K*means and KMSR on the benchmark data sets.

Acc			NMI		Purity	
Data Sets	*K*means	KMSR	*K*means	KMSR	*K*means	KMSR
ecoli	65.50 ± 6.85	**84.56 ± 0.22**	58.70 ± 2.27	**61.47 ± 0.35**	78.46 ± 1.50	**84.56 ± 0.22**
abalone	51.55 ± 0.39	**53.63 ± 0.37**	13.23 ± 0.63	**15.64 ± 0.10**	53.50 ± 0.03	**54.20 ± 0.10**
scale	55.31 ± 5.66	**64.93 ± 0.51**	17.42 ± 8.74	**34.65 ± 1.34**	69.72 ± 5.33	**77.95 ± 1.05**
COIL20	56.06 ± 4.94	**72.81 ± 0.05**	70.51 ± 2.18	**80.26 ± 0.22**	59.64 ± 4.34	**74.55 ± 0.34**
umist	39.51 ± 2.07	**51.04 ± 0.12**	58.33 ± 1.80	**65.09 ± 0.20**	46.51 ± 1.54	**57.39 ± 0.49**
AT&T	52.94 ± 4.21	**63.10 ± 1.80**	73.06 ± 2.51	**78.34 ± 0.50**	61.25 ± 3.41	**67.85 ± 2.00**
YaleB	8.49 ± 0.70	**23.14 ± 0.62**	10.46 ± 1.00	**41.70 ± 0.71**	9.22 ± 0.73	**24.25 ± 0.79**
Yale	39.09 ± 4.61	**44.48 ± 2.04**	45.19 ± 3.61	**49.13 ± 1.60**	41.36 ± 3.92	**46.06 ± 1.29**
PIE	33.56 ± 1.97	**92.38 ± 0.91**	66.40 ± 0.92	**98.13 ± 0.22**	39.22 ± 1.27	**94.45 ± 0.57**
AR	15.25 ± 0.31	**33.48 ± 1.06**	48.43 ± 0.65	**62.38 ± 0.86**	16.18 ± 0.39	**37.40 ± 1.04**
MNIST	52.04 ± 3.16	**56.17 ± 0.31**	49.83 ± 1.93	**50.83 ± 0.00**	55.83 ± 2.63	**57.50 ± 0.00**
jaffe	27.88 ± 2.92	**34.91 ± 1.33**	12.51 ± 3.02	**17.25 ± 1.35**	29.62 ± 3.38	**35.94 ± 1.22**

**Figure 3:**
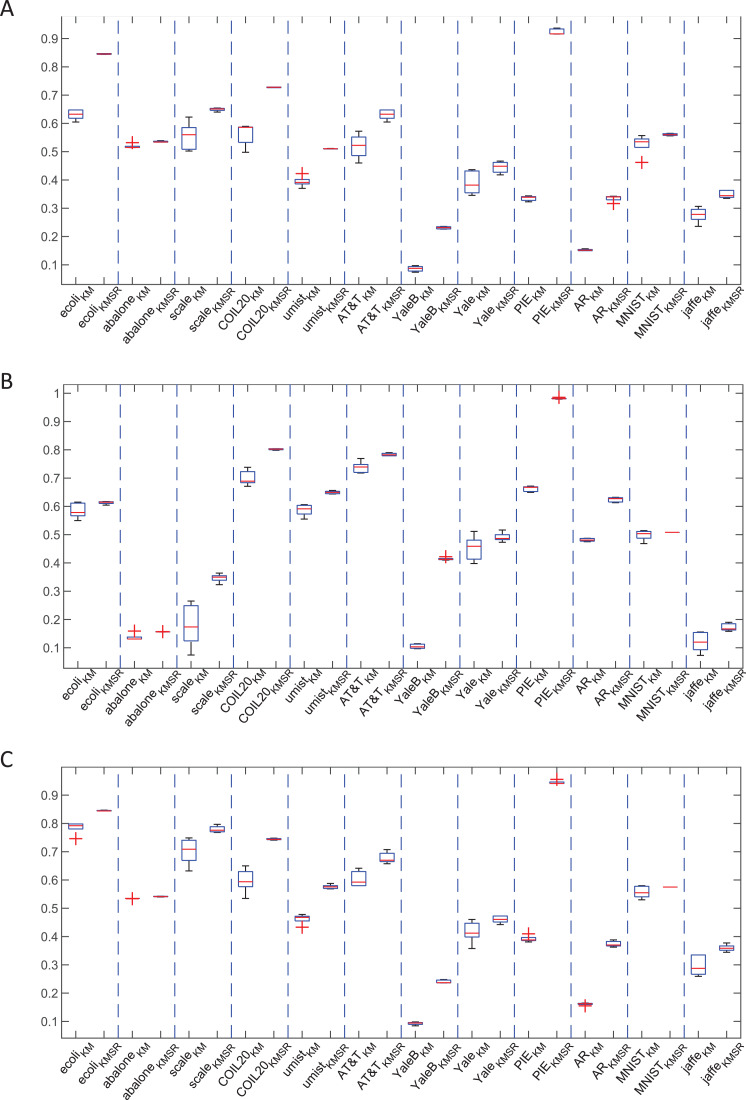
Clustering results obtained by *K*means and KMSR in statistical box diagrams. (A) Acc. (B) NMI. (C) Purity.

Besides the mean values, we can observe that the standard deviations on all the data sets corresponding to the KMSR is much smaller than those of *K*means, which are more explicitly shown by the statistical box digrams in Fig. 3. This means that KMSR is superior to *K*means on the model stability. We think that this improvement comes from the optimization of the orthogonal and normalized rotation matrix instead of the random initialization of cluster centroids in *K*means clustering.

**PART 2**. By constructing the graph affinity matrix in the ‘Heatkernel’ scheme, we show the experimental results of comparing KMSR-G with the four closely related models, NCut+KM, NCut+SR, RCut+KM and RCut+SR, in Table 3 where the best results are highlighted in boldface. It is obvious that KMSR-G obtained better clustering performance than the other models in most cases. For example, KMSR-G performs pretty well on the ecoli, COIL20, AT&T, PIE and MNIST data sets, which respectively obtains the improvements of 7.78%, 3.76%, 2.7%, 12.83%, and 4.05% in comparison with the second-best method in terms of the Acc metric.

**Table 3:** Clustering performance (%) of KMSR-G and related models on twelve data sets.

Acc					
Data Sets	NCut+KM	NCut+SR	RCut+KM	RCut+SR	KMSR-G
ecoli	73.70 ± 0.55	75.26 ± 1.04	75.15 ± 5.25	77.84 ± 1.49	**85.63 ± 0.01**
abalone	49.42 ± 3.85	50.10 ± 2.68	48.87 ± 4.30	50.97 ± 0.00	**51.13 ± 0.22**
scale	65.61 ± 0.31	66.15 ± 0.50	63.37 ± 3.08	65.17 ± 1.61	**66.72 ± 0.45**
COIL20	72.46 ± 4.80	76.58 ± 5.16	71.45 ± 4.76	77.77 ± 3.31	**81.53 ± 0.69**
umist	50.77 ± 2.10	53.97 ± 1.98	51.32 ± 3.86	55.29 ± 1.56	**56.43 ± 1.35**
AT&T	62.70 ± 2.15	66.30 ± 2.53	62.38 ± 1.25	63.46 ± 1.01	**69.00 ± 0.71**
YaleB	32.69 ± 1.19	43.47 ± 0.33	33.62 ± 1.31	**43.57 ± 0.06**	37.76 ± 0.03
Yale	42.55 ± 0.66	42.61 ± 1.73	41.64 ± 1.56	43.42 ± 1.60	**44.24 ± 0.86**
PIE	82.56 ± 3.13	84.93 ± 3.16	81.55 ± 2.07	84.82 ± 3.96	**97.76 ± 0.01**
AR	16.65 ± 0.23	16.80 ± 0.15	16.56 ± 0.26	16.92 ± 0.11	**17.04 ± 0.06**
MNIST	49.29 ± 3.46	50.01 ± 1.45	49.70 ± 2.33	50.75 ± 1.24	**54.80 ± 0.00**
jaffe	24.43 ± 0.60	25.68 ± 0.24	24.62 ± 1.12	25.94 ± 0.48	**25.94 ± 0.00**
NMI					
ecoli	55.94 ± 0.00	56.75 ± 0.47	57.86 ± 1.31	58.37 ± 0.19	**67.55 ± 0.24**
abalone	10.05 ± 4.54	11.42 ± 3.06	9.47 ± 4.54	12.41 ± 0.00	**12.41 ± 0.00**
scale	34.23 ± 0.35	34.64 ± 0.46	32.26 ± 3.88	34.13 ± 1.75	**36.92 ± 0.00**
COIL20	84.50 ± 1.81	87.66 ± 2.39	84.91 ± 2.41	87.78 ± 2.43	**88.89 ± 0.23**
umist	71.14 ± 1.14	72.66 ± 1.17	70.37 ± 2.01	**73.60 ± 0.81**	73.17 ± 0.30
AT&T	79.04 ± 0.80	80.39 ± 1.20	78.95 ± 0.63	79.20 ± 0.55	**81.34 ± 0.20**
YaleB	43.10 ± 0.80	**51.26 ± 0.24**	41.41 ± 0.91	50.00 ± 0.05	44.37 ± 0.09
Yale	49.94 ± 0.73	50.29 ± 1.16	49.18 ± 1.27	50.95 ± 1.00	**51.71 ± 0.04**
PIE	94.67 ± 1.08	95.12 ± 1.10	93.97 ± 0.97	95.03 ± 1.41	**99.40 ± 0.01**
AR	51.84 ± 0.22	**52.87 ± 0.18**	50.59 ± 0.38	52.11 ± 0.28	52.12 ± 0.32
MNIST	53.78 ± 2.21	55.47 ± 0.95	52.68 ± 1.12	53.69 ± 1.11	**57.03 ± 0.06**
jaffe	8.71 ± 0.82	9.40 ± 0.22	9.48 ± 0.88	9.69 ± 0.40	**11.31 ± 0.66**
Purity					
ecoli	74.68 ± 0.14	75.72 ± 0.61	77.91 ± 0.29	78.12 ± 0.29	**85.63 ± 0.01**
abalone	50.54 ± 4.42	51.93 ± 3.31	49.89 ± 4.90	53.00 ± 0.00	**53.00 ± 0.00**
scale	78.74 ± 0.38	78.91 ± 0.55	77.41 ± 1.35	78.17 ± 0.73	**79.76 ± 0.11**
COIL20	75.23 ± 4.13	81.32 ± 5.24	76.56 ± 4.05	82.67 ± 3.33	**84.20 ± 0.34**
umist	61.96 ± 2.12	63.31 ± 1.97	60.16 ± 3.21	64.33 ± 1.66	**65.04 ± 0.00**
AT&T	66.00 ± 1.79	68.53 ± 2.86	65.75 ± 1.32	67.90 ± 1.02	**72.63 ± 0.53**
YaleB	34.72 ± 1.03	45.28 ± 0.35	35.65 ± 1.04	**45.36 ± 0.07**	39.08 ± 0.03
Yale	42.91 ± 0.51	43.00 ± 1.14	42.52 ± 1.78	43.42 ± 1.60	**44.55 ± 1.29**
PIE	85.51 ± 2.82	85.53 ± 3.24	82.46 ± 2.53	85.46 ± 3.96	**98.39 ± 0.01**
AR	17.29 ± 0.23	17.32 ± 0.13	17.50 ± 0.25	17.56 ± 0.17	**17.71 ± 0.06**
MNIST	55.74 ± 2.62	58.00 ± 2.81	55.55 ± 1.70	57.55 ± 1.59	**61.95 ± 0.07**
jaffe	24.65 ± 0.88	25.68 ± 0.24	24.79 ± 1.36	26.44 ± 0.50	**27.83 ± 0.75**

**Figure 4:**
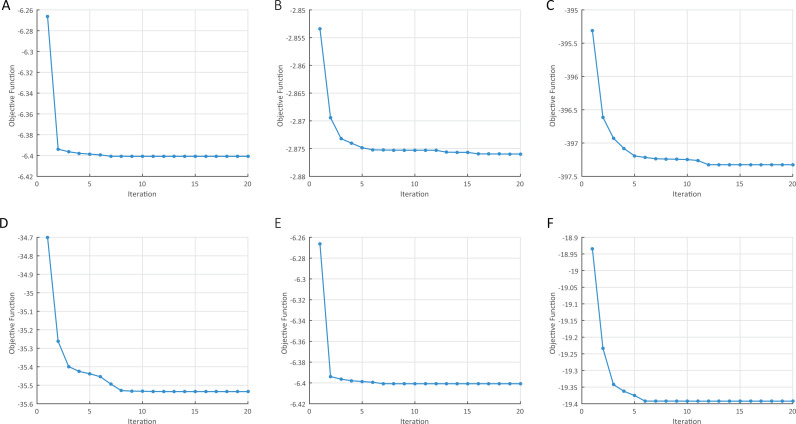
The decreasing of the KMSR-G objective function values in terms of iterations (*λ* = 0.1). (A) abalone. (B) scale. (C) PIE. (D) YaleB. (E) jaffe. (F) umist.

Besides the theoretical analysis on the convergence of KMSR-G in ‘Model complexity and convergence analysis’, , we empirically show the decreasing of its objective function values on the six data sets of abalone, scale, umist, YaleB, PIE and jaffe in Fig. 4. All the results are obtained when the regulation parameter *λ* is set as 10^−1^. We can find that KMSR-G has desirable convergence property and usually converges in a few iterations.

**Figure 5:**
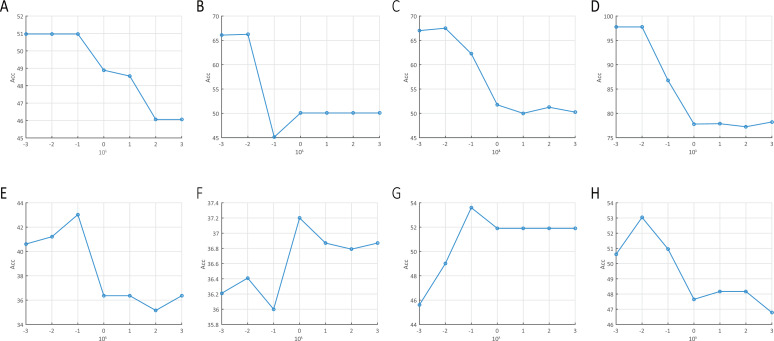
The accuracy of KMSR-G obtained in different settings of *λ*. (A) abalone. (B) scale. (C) AT&T. (D) PIE. (E) Yale. (F) YaleB. (G) MNIST. (H) umist.

In the description of experimental settings, we mentioned that the regulation parameter *λ* is tuned from candidate values {10^−3^, 10^−2^, …, 10^3^}. Here we explore the impact of such regulation parameter on the clustering performance of KMSR-G. We show the clustering accuracy of KMSR-G with the variation of parameter *λ* on the eight of our used data sets in Fig. 5. Generally, it depicts that KMSR-G prefers a small value of *λ* to achieve better clustering accuracy.

**Figure 6:**
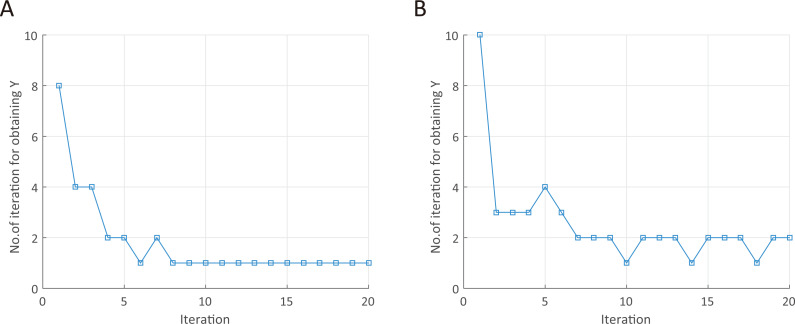
Number of iterations in updating **Y** on AT&T and COIL20 data sets. (A) AT&T. (B) COIL20.

In the optimization of the binary cluster indicator matrix **Y**, it is also in an iterative manner (i.e., the inner loop in Algorithm 2). Here taking the AT&T and COIL20 data sets for example, we show the number of iterations in updating **Y** in Fig. 6. Since the number of clusters is usually small in respective data sets, the updating of **Y** could be a very fast process, usually less than 10 iterations.

Besides the experiments on the rule-based graph (i.e., ‘Heatkernel’ function), we further try two learning-based graphs which are the CAN and PCAN (Nie, Wang & Huang, 2014). CAN can adaptively learn the graph affinity matrix from data by simultaneously considering the non-negativity, normalization and rank constraint (Peng et al., 2020) properties of a desirable graph. PCAN is its projected version, which takes the three-fold constraints into account in a subspace. We present the clustering results of CAN, KMSR-GC, PCAN and KMSR-GPC in Table 4. From the obtained results, we have the following two findings.

**Table 4:** Clustering performance (%) of KMSR-G based on learning-based graphs of CAN and PCAN.

Data Sets	CAN	KMSR-GC	PCAN	KMSR-GPC
Acc				
ecoli	81.96	**81.96 ± 0.00**	81.96	**81.96 ± 0.00**
abalone	50.97	**53.08 ± 0.00**	50.97	**51.59 ± 0.88**
scale	53.28	**63.20 ± 0.00**	66.72	**67.84 ± 0.01**
COIL20	83.96	**86.01 ± 0.05**	81.81	**81.81 ± 0.00**
umist	69.04	**71.13 ± 0.30**	54.78	**55.04 ± 0.37**
AT&T	55.25	**63.00 ± 1.77**	60.00	**61.50 ± 0.01**
YaleB	37.12	**37.12 ± 0.00**	38.69	**38.69 ± 0.00**
Yale	41.21	**42.42 ± 0.86**	40.00	**40.91 ± 0.43**
PIE	100.0	**100.0 ± 0.00**	100.0	**100.0 ± 0.00**
AR	13.67	**13.96 ± 0.06**	13.33	**13.63 ± 0.06**
MNIST	45.80	**49.37 ± 2.14**	45.80	**45.80 ± 0.00**
jaffe	22.64	**27.59 ± 0.33**	25.00	**26.18 ± 1.00**
NMI				
ecoli	65.74	**65.74 ± 0.00**	66.32	**66.32 ± 0.00**
abalone	12.49	**12.52 ± 0.00**	12.41	**12.42 ± 0.00**
scale	17.80	**18.42 ± 0.00**	17.04	**17.17 ± 0.01**
COIL20	91.34	**92.49 ± 0.01**	89.60	**89.60 ± 0.00**
umist	81.72	**82.15 ± 0.60**	65.02	**65.02 ± 0.00**
AT&T	73.88	**78.82 ± 0.85**	74.13	**77.32 ± 0.22**
YaleB	39.98	**39.98 ± 0.00**	39.99	**40.22 ± 0.33**
Yale	44.95	**45.77 ± 0.20**	42.64	**43.06 ± 0.58**
PIE	100.0	**100.0 ± 0.00**	100.0	**100.0 ± 0.00**
AR	42.93	**43.04 ± 0.01**	35.88	**38.04 ± 0.02**
MNIST	49.87	**49.87 ± 0.00**	46.35	**46.46 ± 0.00**
jaffe	10.80	**11.28 ± 0.56**	11.10	**11.94 ± 0.83**
Purity				
ecoli	82.87	**82.87 ± 0.00**	82.87	**82.87 ± 0.00**
abalone	53.08	**53.00 ± 0.00**	53.00	**53.00 ± 0.00**
scale	67.20	**67.20 ± 0.00**	70.08	**70.24 ± 0.01**
COIL20	87.22	**88.26 ± 0.00**	86.11	**86.11 ± 0.00**
umist	74.78	**76.00 ± 0.74**	62.78	**62.78 ± 0.00**
AT&T	64.25	**68.38 ± 1.24**	67.50	**68.25 ± 0.01**
YaleB	39.73	**39.73 ± 0.00**	40.56	**40.56 ± 0.00**
Yale	43.03	**44.85 ± 0.00**	42.42	**42.73 ± 0.43**
PIE	100.0	**100.0 ± 0.00**	100.0	**100.0 ± 0.00**
AR	16.83	**16.96 ± 0.06**	17.17	**17.25 ± 0.01**
MNIST	50.90	**50.90 ± 0.00**	51.10	**51.10 ± 0.00**
jaffe	23.11	**28.07 ± 0.33**	25.94	**26.89 ± 0.67**

• By comparing the results in Tables 3 and 4, we can find that KMSR-G with CAN (or PCAN) obtained superior performance than that with ‘Heatkernel’ function in most cases. This indicates that the graph quality is the leading factor in graph-based clustering models. Even for one graph construction method, it functions differently on different data sets according to our experimental results.
• We can generally declare that KMSR-GC is better than its baseline method CAN; and similarly, KMSR-GPC outperforms PCAN on all the data sets. Though CAN and PCAN jointly performs graph learning and clustering, the indicator matrices learned by them are still real-valued ones; therefore, the postprocessing step is necessary to make discretization. This limitation is avoided in our proposed KMSR-G model and thus improved performance is obtained.

## Conclusion

In this paper, based on the connection between *K*means clustering and spectral clustering, we proposed a new *K*means formulation by jointly performing spectral embedding and spectral rotation. The formulated KMSR model can not only improve the clustering performance but also enhance the model stability in comparison with the traditional *K*means clustering. Further, the KMSR model was generalized as KMSR-G which can take any pre-defined graph as input and output the final discrete cluster indicator matrix. An efficient method in coordinate blocking framework was designed to optimize the proposed KMSR (KMSR-G) model objective. Extensive experiments were conducted on representative data sets to show the effectiveness of the proposed models in data clustering. As our future work, we will consider unifying the three components of graph construction, spectral embedding and spectral rotation in graph-based clustering into a complete framework. That is, we will incorporate the graph learning process into the present KMSR-G model.

##  Supplemental Information

10.7717/peerj-cs.450/supp-1 Supplemental Information 1 Source code for our proposed KMSR-G model

10.7717/peerj-cs.450/supp-2 Supplemental Information 2 Abalone, AR, AT&T, COIL20, ecoli, jaffe, MNIST and PIE data

10.7717/peerj-cs.450/supp-3 Supplemental Information 3 Scale, umist, Yale and YaleB data

10.7717/peerj-cs.450/supp-4 Supplemental Information 4 Data

## Additional Information and Declarations

### Data Availability

The following information was supplied regarding data availability: Data and code are available in the Supplementary Files and GitHub: https://github.com/finepix/KMSR-G.git.
